# From Field to Fundus: A Peripheral Retinal Tear Following Football-Related Ocular Trauma

**DOI:** 10.7759/cureus.109005

**Published:** 2026-05-17

**Authors:** Muhammad Hafiz As-Shaarani Mohd Amin, Adzura Salam

**Affiliations:** 1 Ophthalmology, International Islamic University Malaysia, Kuantan, MYS

**Keywords:** commotio retinae, conjunctival laceration, laser retinopexy, retinal tear, sports-related ocular trauma

## Abstract

Sports-related ocular trauma is an important cause of preventable visual morbidity, particularly in contact sports such as football (soccer). Although many injuries initially present with mild anterior segment findings, significant posterior segment pathology may coexist and can be easily overlooked. We report the case of a 17-year-old male football player who presented with left eye pain, blurred vision, and periorbital bleeding following blunt ocular trauma during a football match. Examination revealed traumatic mydriasis with anterior uveitis, subconjunctival hemorrhage with conjunctival laceration and exposed Tenon's tissue, as well as commotio retinae with a peripheral horseshoe-shaped retinal tear. No retinal detachment was identified. The patient underwent prompt laser retinopexy and received topical anti-inflammatory and antibiotic therapy, resulting in favorable anatomical and visual outcomes. This case highlights the importance of maintaining a high index of suspicion in sports-related ocular trauma, as relatively subtle anterior segment findings may mask significant posterior segment injury. Careful and systematic anterior and posterior segment examination, including peripheral retinal evaluation, is essential to avoid missing sight-threatening complications and to ensure timely intervention.

## Introduction

Ocular trauma remains a significant concern in sports, with presentations ranging from minor surface injuries to vision-threatening pathology. Blunt ocular impact, particularly in contact sports such as football (soccer), can transmit force across the globe and result in combined anterior and posterior segment involvement. It is estimated that sports-related eye injuries account for approximately 30,000 emergency department visits annually in the United States, in which eye injuries were the primary diagnosis in more than 70% of cases [1]. Football is a significant contributor to these injuries [2,3]. Despite this, a large proportion of these injuries are preventable with appropriate protective measures [4]. Clinically, anterior segment findings such as subconjunctival hemorrhage and traumatic uveitis are frequently encountered and may appear relatively benign. However, these findings can mask more significant posterior segment pathology, especially when the mechanism involves blunt force transmission. The coexistence of conjunctival laceration with exposed Tenon's tissue and a peripheral retinal tear is uncommon and may present a diagnostic challenge, particularly if posterior segment examination is not performed meticulously [5,6]. Failure to identify such injuries early may lead to delayed complications, including retinal detachment and permanent visual impairment. This report describes a case of combined anterior and posterior segment injury following a football-related blunt trauma, highlighting the importance of early assessment and meticulous ocular examination to prevent sight-threatening complications.

## Case presentation

A 17-year-old previously healthy male student-athlete from a football academy presented to the emergency department with left eye pain, blurred vision, tearing, and periorbital bleeding following a collision with an opponent during a football match. He reported an immediate onset of symptoms after sustaining a direct impact to the left eye and falling to the ground. There was no history of loss of consciousness, headache, vomiting, floaters, flashes, or diplopia. He had no prior history of ocular trauma.

**Figure 1:**
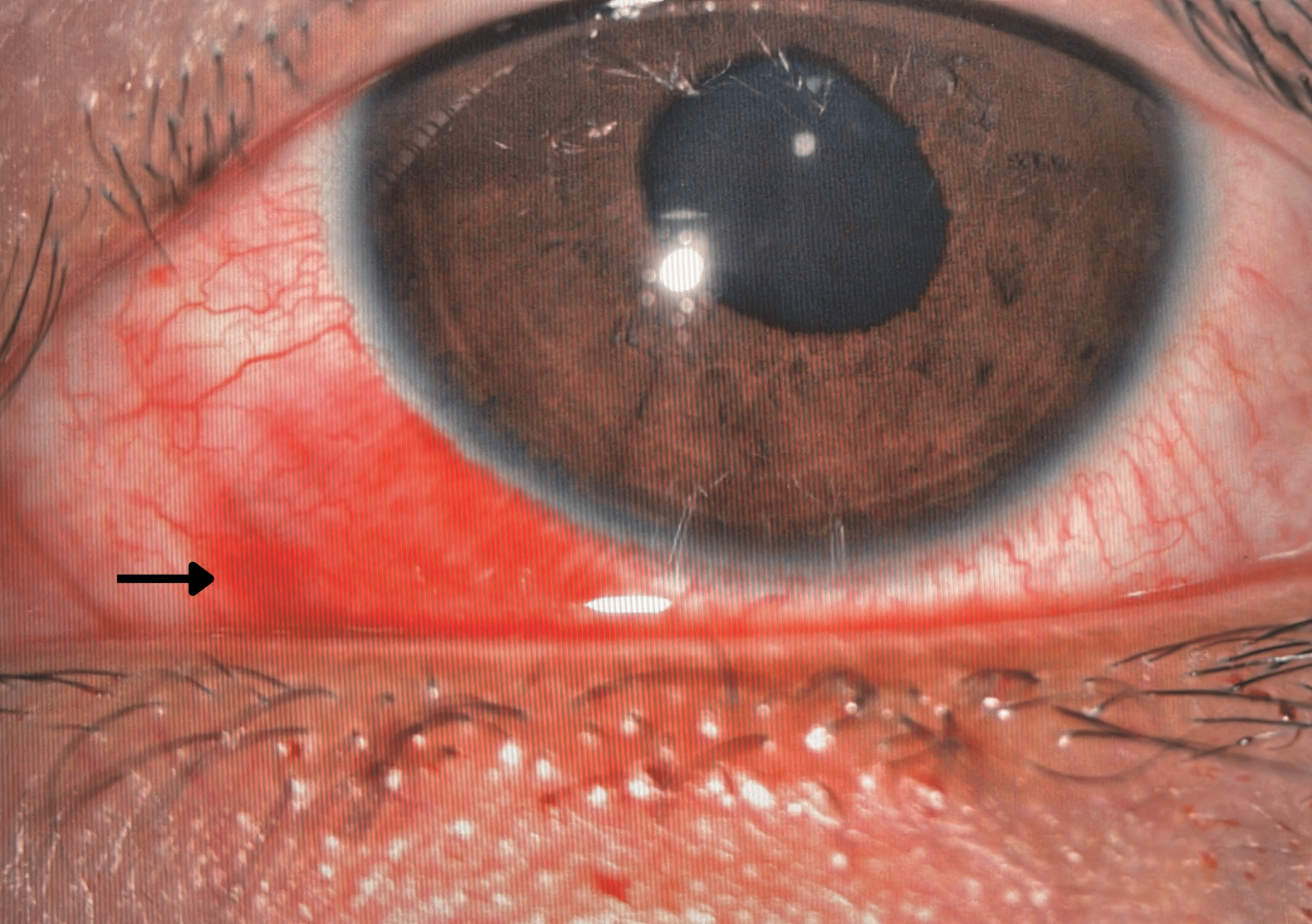
Anterior segment image of the left eye highlighting the subconjunctival hemorrhage (black arrow) and dilated pupil

**Figure 2:**
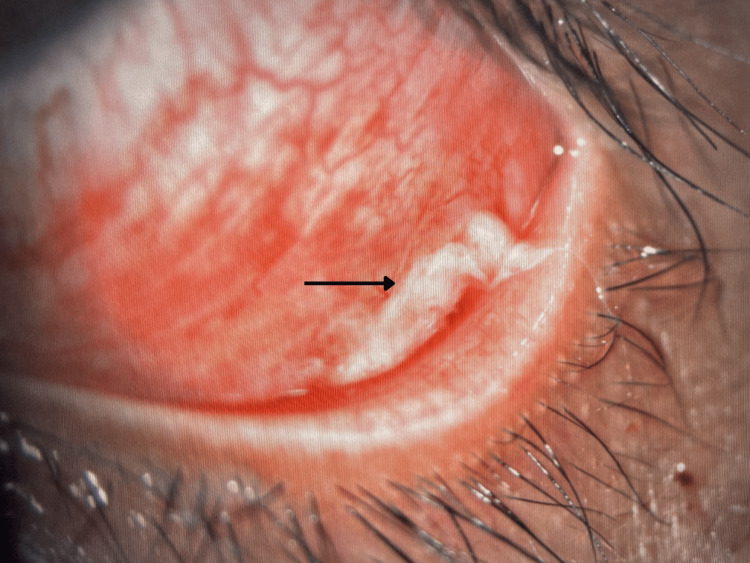
Anterior segment image of the left eye showing an exposed Tenon's tissue (black arrow) from the lateral part of the conjunctival fornix

On examination, visual acuity was 6/18 in the left eye, improving to 6/12 with pinhole, and 6/6 in the right eye. Anisocoria was present, with a 5 mm pupil in the left eye compared to 2 mm in the right eye. Reverse relative afferent pupillary defect (RAPD) was negative. Periorbital examination showed a superficial abrasion over the upper eyelid, associated with swelling, mild mechanical ptosis, and inferior eyelid ecchymosis. Orbital palpation demonstrated no step deformity or crepitus. Intraocular pressure was within normal limits in both eyes. Ocular motility testing demonstrated a restricted movement in the left eye with pain on adduction and depression. Slit-lamp examination of the left eye demonstrated subconjunctival hemorrhage with exposed Tenon's tissue and underlying hematoma at the inferonasal region (Figures 1-2).

**Figure 3:**
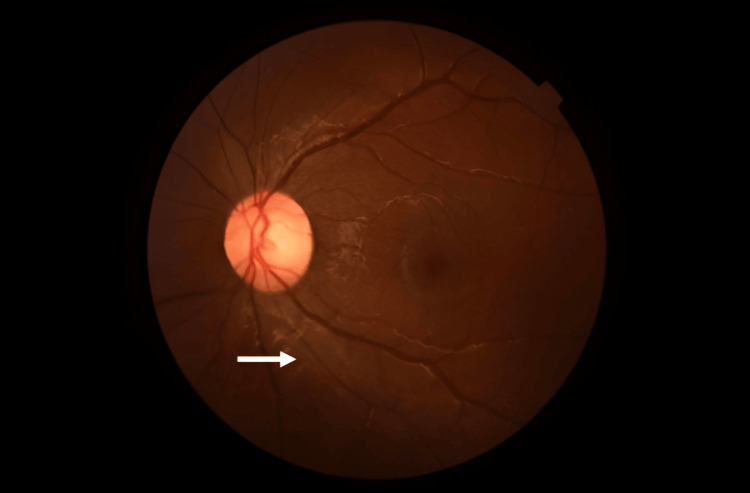
A fundus photograph of the left eye showing the commotio retinae near the inferotemporal vessels (white arrow)

**Figure 4:**
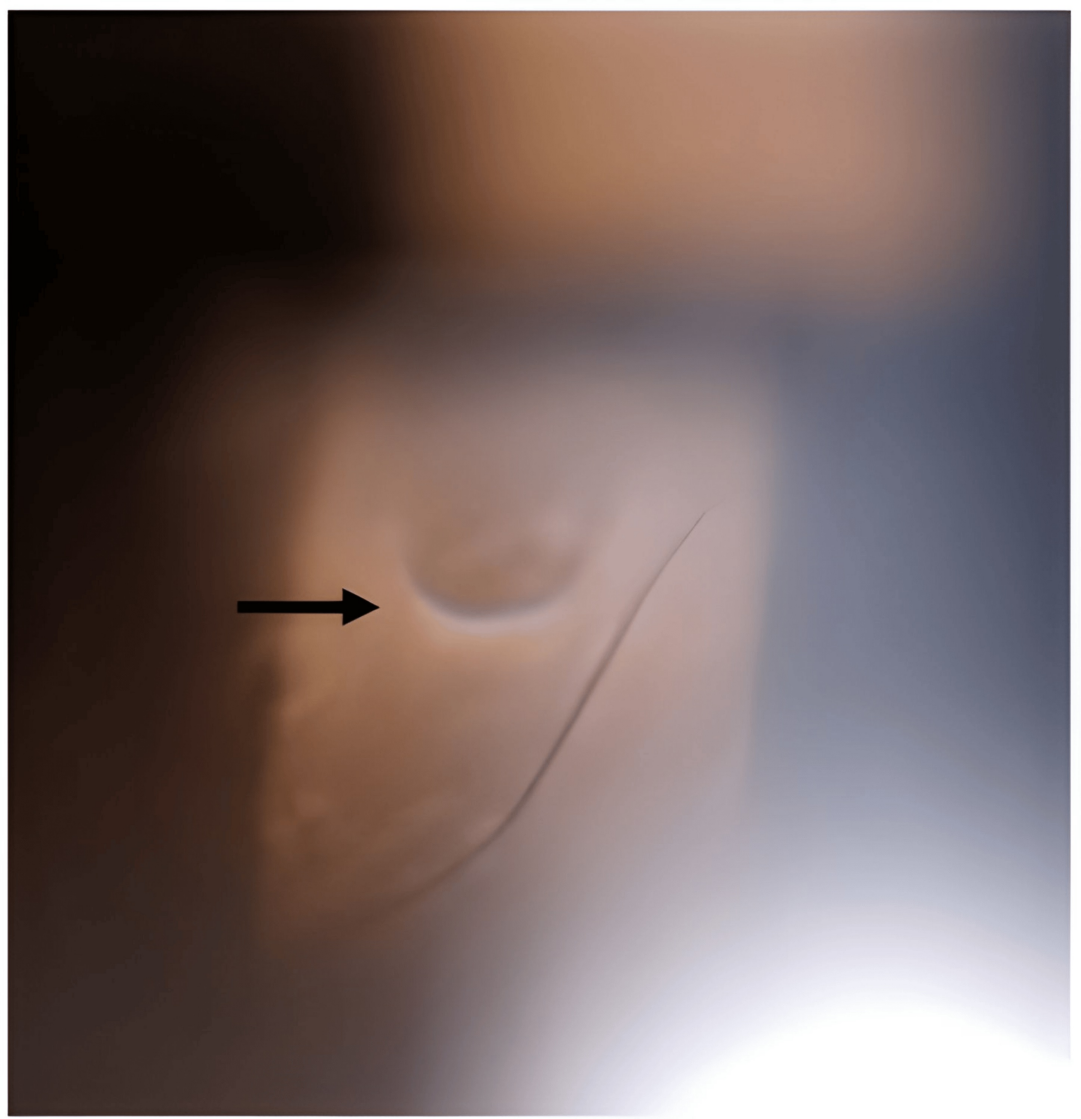
A detailed fundus view obtained using a slit-lamp biomicroscope with a diagnostic lens. The retinal tear (black arrow) is visible in the peripheral retina, located at the 6 o'clock position. The tear appears as a horseshoe-shaped break with sharp, irregular edges

The cornea was clear with minimal superficial punctate epithelial erosion (SPEE), and Seidel's test was negative. The anterior chamber was deep with 1+ cells and pigments. The pupil was sluggish and mid-dilated, consistent with traumatic mydriasis. The crystalline lens was clear and well-positioned. Fundus examination revealed a pink optic disc with a cup-to-disc ratio of 0.3-0.4 and a flat macula. There was commotio retinae noted near the inferotemporal vessels, along with a peripheral horseshoe-shaped retinal tear at the 6 o'clock position (Figures 3-4). No vitreous hemorrhage, choroidal hemorrhage, or retinal detachment was observed. 

**Figure 5:**
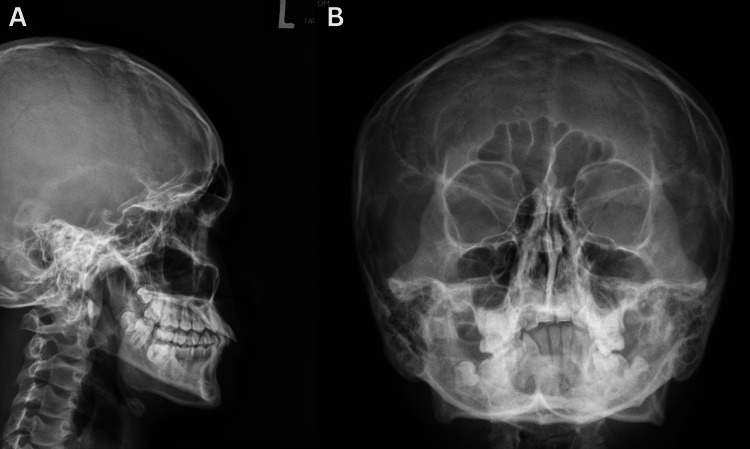
Skull radiographs showing no obvious orbital wall fracture: (A) lateral skull radiograph and (B) occipitomental (Waters view) radiograph

**Figure 6:**
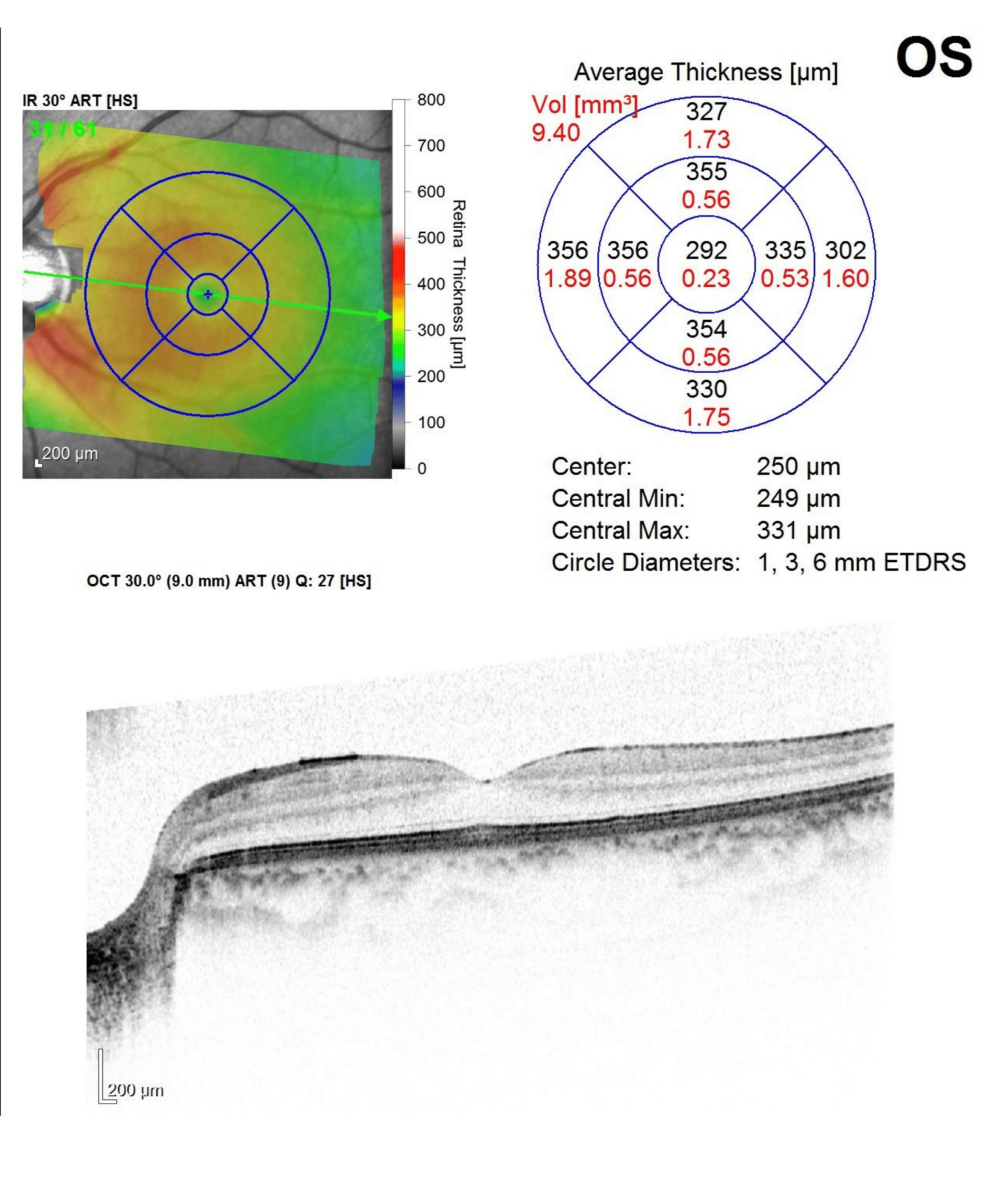
Optical coherence tomography demonstrating a preserved foveal contour without intraretinal fluid, subretinal fluid, or macular hole

The right eye examination was unremarkable. Skull and occipitomental (Waters view) X-rays showed no evidence of orbital wall fracture (Figure 5). Optical coherence tomography (OCT) of the macula was normal (Figure 6).

A diagnosis of left eye traumatic mydriasis with uveitis, commotio retinae with horseshoe-shaped retinal tear, and conjunctival laceration with exposed Tenon's tissue was made. The patient was treated with topical dexamethasone 0.1%, topical chloramphenicol 0.5%, and oral paracetamol. Laser retinopexy was performed to surround the retinal tear. At the three-day follow-up, the patient showed significant clinical improvement, with visual acuity improving to 6/9 unaided and 6/6 with pinhole. There was a reduction in pain, periorbital swelling, and anisocoria. Fundus examination confirmed adequate laser retinopexy scars, and the patient was continued on tapering topical therapy.

## Discussion

Sports-related ocular trauma remains an important cause of preventable visual morbidity, particularly in contact sports such as football, where blunt impact to the eye is common [1-3]. Although many cases initially present with apparently mild anterior segment findings, this case highlights the potential for significant concurrent posterior segment injury that may be easily overlooked.

Blunt trauma to the globe results in sudden anteroposterior compression with equatorial expansion, generating vitreoretinal traction forces. These forces predispose to peripheral retinal breaks, particularly horseshoe-shaped tears [7]. This explains the concurrence of commotio retinae and retinal tear observed in this patient. Importantly, these posterior segment injuries may not correlate with the severity of external findings, and reliance on anterior segment findings alone may lead to the underestimation of the extent of trauma.

A key learning point from this case is the critical importance of early and careful ocular assessment. Initial anterior segment findings such as conjunctival laceration with exposed Tenon's tissue, traumatic mydriasis, and uveitis should prompt clinicians to consider the possibility of globe-wide injury rather than isolated superficial trauma. These findings can mask deeper structural damage, particularly when the mechanism involves direct blunt impact. This should prompt a systematic and comprehensive ocular examination, including careful evaluation of the peripheral retina. Without a thorough dilated fundus examination, small peripheral retinal tears, especially those located inferiorly, can be easily missed.

Undetected retinal tears carry a significant risk of progression to rhegmatogenous retinal detachment, a potentially sight-threatening complication requiring surgical intervention [8]. Early detection allows timely treatment with laser retinopexy which has been shown to be safe and effective in preventing progression to retinal detachment [9]. In this case, prompt intervention resulted in a favorable anatomical and visual outcome.

Nevertheless, other findings in this case also require specific attention and intervention. Follow-up care showed significant improvement, emphasizing the need for ongoing monitoring and adjustment of treatment as needed. High-risk sports, particularly contact sports like football, could become significantly safer with the broad implementation of eye protection and safer rules [4,10].

## Conclusions

This case highlights that sports-related ocular injury can result in significant posterior segment pathology despite relatively mild anterior segment findings. Clinicians should maintain a high index of suspicion and perform a comprehensive anterior and posterior segment evaluation in all cases of ocular trauma. Peripheral retinal tears, particularly those located inferiorly, may be easily missed without a careful and systematic examination. Early recognition and timely treatment with laser retinopexy are essential in preventing progression to retinal detachment and preserving visual outcomes. Greater awareness of sports-related ocular injuries, together with preventive measures such as protective eyewear in high-risk sports, may help reduce the incidence of such injuries.

## References

[1] Haring RS, Sheffield ID, Canner JK, Schneider EB (2016). Epidemiology of sports-related eye injuries in the United States. JAMA Ophthalmol.

[2] Drolsum L (1999). Eye injuries in sports. Scand J Med Sci Sports.

[3] Capao Filipe JA, Fernandes VL, Barros H, Falcao-Reis F, Castro-Correia J (2003). Soccer-related ocular injuries. Arch Ophthalmol.

[4] Heimmel MR, Murphy MA (2008). Ocular injuries in basketball and baseball: what are the risks and how can we prevent them?. Curr Sports Med Rep.

[5] Jones NP (1989). Eye injury in sport. Sports Med.

[6] Filipe JA, Barros H, Castro-Correia J (1997). Sports-related ocular injuries: a three-year follow-up study. Ophthalmology.

[7] Cox MS (1980). Retinal breaks caused by blunt nonperforating trauma at the point of impact. Trans Am Ophthalmol Soc.

[8] Neumann E, Hyams S (1972). Conservative management of retinal breaks. A follow-up study of subsequent retinal detachment. Br J Ophthalmol.

[9] Somoskeoy T, Shah P (2021). Safety and efficacy of the use of navigated retinal laser as a method of laser retinopexy in the treatment of symptomatic retinal tears. Eye (Lond).

[10] Capão Filipe JA, Rocha-Sousa A, Falcão-Reis F, Castro-Correia J (2003). Modern sports eye injuries. Br J Ophthalmol.

